# Self-reported continuing professional development needs of medical laboratory professionals in Ghana

**DOI:** 10.1186/s12960-023-00859-9

**Published:** 2023-09-12

**Authors:** Mainprice Akuoko Essuman, Nii Armah Addy, Samuel Essien-Baidoo, Irene Esi Donkoh, Felix A. Botchway, Justice Afrifa, Prince Agyeman, Leticia Awontayami Amaama, Samuel Amoah, Felix B. K. Sorvor, Richard K. D. Ephraim

**Affiliations:** 1 Department of Medical Laboratory Science, School of Allied Health Sciences, College of Health and Allied Sciences, University of Cape Coast, Cape Coast, Ghana; 2 Institute of Leadership and Management in Education (InLaME), Accra, Ghana; 3 Department of Medical Laboratory Technology, Accra Technical University, Accra, Ghana; 4 School of Public Health, University of Ghana, Legon, Ghana; 5 Laboratory Department, University Health Services, University of Cape Coast, Cape Coast, Ghana; 6 National Tuberculosis Control Programme, Korle Bu, Accra, Ghana

**Keywords:** Continuing professional development, Medical laboratory professionals, Healthcare, Online survey, Training needs

## Abstract

**Background:**

Because of the essential nature of the work of medical laboratory professionals, continuing development in knowledge and skills is indispensable. The study aimed at identifying and prioritizing the development and training needs of medical laboratory professionals in Ghana. This is expected to help in developing focused continuing professional development (CPD) that meets the needs of practitioners as well as the changing medical trends.

**Methods:**

An online cross-sectional survey in February 2022 using a structured questionnaire was conducted. Respondents were asked questions that collected demographic and work-related data about them, their participation, preference, and challenges in being part of CPDs. Finally, a list of topics based on (i) quality management systems, (ii) technical competence, (iii) laboratory management, leadership, and coaching, (iv) pathophysiology, and (iv) data interpretation and research were asked with the option to rate them on a 3-point scale (most, moderate, and least) in order of importance.

**Results:**

A total of 316 medical laboratory professionals participated in the study. Overall, the most frequently selected topics for training based on domains for CPD training and ranking as most important were (i) quality management systems, (mean = 80.59 ± 9.024; 95% CI = 73.04–88.13); (ii) pathophysiology, data interpretation, and research (mean = 78.0 ± 6.973; 95% CI = 73.97–82.03); (iii) technical competence (mean = 73.97 ± 10.65; 95% CI = 66.35–81.59); and (iv) laboratory management, leadership, and coaching (mean = 72.82 ± 9.719; 95% CI = 67.44–78.2). The factors affecting the choice of training needs included the medical laboratory professionals’ current place of work, years in service, the reason for attending CPD activities, the period for attending the last CPD, being in a supervisory role, and the number of staff being supervised. Face-to-face presentations, training workshops, and hands-on workshops were the most preferred modes of CPD delivery with financial implications and workload/time constraints being the main challenges impeding CPD participation.

**Conclusion:**

The identified needs will help in developing CPD programs that address what medical laboratory professionals prioritize as training needs. Stakeholders should incorporate these training needs into future programs and address the challenges highlighted in this study to have more relevant training for medical laboratory professionals.

## Introduction

Advances in global health and medicine mainly due to the emergence of new diseases, the discovery of advanced diagnostic techniques and facilities, and increased demand for laboratory services necessitate that medical professionals consistently maintain their competencies more than ever [1, 2]. It is therefore noteworthy that a pragmatic review of the knowledge and skills needs of medical professionals including medical laboratory professionals (MLPs) must be a rational precursor to the development of any training program directed at solving these needs [3, 4].

Continuing professional development (CPD) embodies a lifelong process of active participation in learning activities that assist in developing and maintaining continuing competence, enhancing professional practice, and supporting the achievement of career goals [5]. CPD activities can take the form of traditional classroom face-to-face sessions to online learning with the same goal of creating a qualified workforce capable of successfully meeting the needs of the populations they serve [6].

Because of the enormous benefits CPD has on patients care and the professional development of health workers, it is important to promote the continuing professional development of health workers [7–9]. CPD contributes to improving the quality of care and health outcomes, such as reducing the likelihood of patients dying or the odds of failure to rescue them [10]. It also promotes motivation, commitment, and satisfaction among professionals [9] and, as a consequence, their retention and performance [7]. Like many other countries [3, 11, 12], CPD is a mandatory requirement for the renewal of a license to practice as a medical laboratory professional in Ghana. When not properly structured to meet the needs of workers and patients, it would be difficult to derive the needed benefit CPD is supposed to have on our health care system.

It is reported that self-motivation, relevance to practice, preference for workplace learning, strong enabling leadership, and positive workplace culture are key factors that optimize the impact of CPD among health workers [13]. Meanwhile, in the rapidly changing healthcare context, it is reported that health workers participate in CPD when they have reasons to do so [14]. It is key, therefore, that strategies aimed at promoting the participation of health workers in CPD address their real needs [10]. Human resource needs are said to revolve around existing and expected professional practice requirements, enabling competence and capacities, and learning and change requirements [4]. Unfortunately, the strategies designed to promote health workers’ CPD do not always consider these needs [15, 16].

It is well-accepted that current trends in healthcare such as the emergence of COVID-19 have exposed loopholes in our healthcare system [1, 2], not only in the lack of medical facilities but in the gap in knowledge and skills. It follows then, that a pragmatic review of the knowledge and skills needs of MLPs must be a rational precursor to the development of any training program [3, 4]. Moreover, if a reliable occupational profile of MLPs could be obtained, then there would be a sound foundation on which to identify and define the core competencies, which would form the basis of basic laboratory education in a variety of local contexts to meet current trends. These could be further used to inform national and regional education curricula and care standards, accredit topics for future CPDs, and consequent integration into our mainstream healthcare education. It is similarly essential to identify skill deficits and underperformance in existing roles so that CPD can be devised in a manner that would directly address areas of suboptimal clinical performance [3].

Given the above information, self-reported training needs and experiences of MLPs regarding their CPD may provide insight into the complexity of MLPs’ involvement (or lack thereof) in CPD. This would also help identify their needs to consider when designing programs that realistically support CPD. To this end, this study was undertaken to highlight the challenges the Ghanaian MLP faces in accessing CPDs, and to identify and prioritize the training requirements of medical laboratory professionals in Ghana. Additionally, we also explored the factors that are associated with training needs prioritization by laboratory professionals. This would help to develop a focused and systematic continuing professional development module that meets the needs of practitioners.

## Methods

### Study design

A cross-sectional survey was employed and used an online questionnaire survey tool to reach MLPs across the country. A well-structured survey tool designed using google forms was delivered online over 20 days (3rd to 23rd February 2022).

### Study population

The target population in this study included MLPs working in health care settings (government, private or quasi-government), non-governmental organizations, regulatory organizations, research, and academic institutions in Ghana. The inclusion criteria were full- or part-time MLPs with a license from the Allied Health Professions Council—the main regulatory body for training of medical laboratory personnel, issuance and renewal of licenses, and approval of CPDs. Participants were required to have a minimum of one year of post-qualification working experience at the time of the study.

### Sampling technique

Snowball sampling, a nonprobability sampling approach that samples clusters of connected participants, was used to recruit participants. The recruitment of participants was performed online, mainly through WhatsApp platforms. Because of the possibility of having people who do not meet eligibility criteria filling the forms, the forms were not shared on Facebook, Twitter, and other open-spaced social networking platforms. The use of online surveys is described to be an innovative technique with the advantage of not only being cost-effective and time efficient but being able to enable access to large and geographically distributed populations [17]. The study instrument used for the study was user-friendly, participant-specific, and ethically sensitive with multiple responses being avoided in line with earlier recommendations [17]. Initially, some laboratory professionals who were administrators for WhatsApp pages of various laboratory groups were conveniently identified by the research team. These administrators were contacted and provided with information about the study, including the survey link, and were asked to share the link on their platforms. In addition, the researchers conveniently created a broadcast list of eligible laboratory professionals on their contact and disseminated messages to them to complete the forms and circulate to other laboratory professionals they know.

### Questionnaire development and survey process

The survey questionnaire and its content were developed based on a review of studies on similar subject [3, 18] with some modifications to suit the context of medical laboratory practice in Ghana. To get a desirable outcome, the questionnaire was shared among immediate colleagues as the target audience to scrutinize the accessibility, clarity, and relevance of the survey questions.

The first section of the questionnaire elicited general information about participants and their professional practice. Participants were asked to provide their age, gender, cadre in the medical laboratory profession, number of years they have worked, and the location of their current place of work. Cadre type in medical laboratory practice was reported in terms of academic qualification as laboratory assistant (2-year certificate), technician (3-year diploma), or scientist (4/6-year bachelor’s degree). Participants were asked to indicate whether they are in a supervisory role and if answered “Yes” asked to provide the number of personnel under their supervision.

Questions were asked to elicit information about participants’ participation in CPDs. First, participants were asked to indicate the last time they attended a CPD event from three options (less than 1 year, 1–3 years, or more than 3 years) and to indicate their main reason for participating in CPD events. For this, they were made to choose among three options (to maintain and improve knowledge and skills, to facilitate license renewal, and to interact and exchange expertise with colleagues). Furthermore, participants were asked to select their preferences for delivery of CPD activities from a list of seven options, namely face-to-face presentations, live video conferences, hands-on workshops, journal club at the workplace, training workshops, and directed learning in the workplace followed by a quiz and internet-based learning. Participants were also asked to indicate the challenges they face in attending CPD training from 6 options provided with the chance to indicate any other challenge which was not listed.

The study instrument used to assess the study needs of participants included 46 items organized into 5 key domains related to laboratory practice, namely (i) quality management systems (8 items); (ii) technical competence (10 items); (iii) laboratory management, leadership, and coaching (15 items); (iv) pathophysiology, data interpretation and research (14 items). Participants were asked to rank their self-perceived training needs using a 3-point scale in order of importance (most, moderate, and least).

### Data processing and analysis

Data collected from the online survey were first entered into Microsoft Excel, double-checked, and analyzed using IBM SPSS version 26.0 (Statistical Package for the Social Sciences, Chicago, IL USA) with GraphPad Prism 8 (GraphPad Software, San Diego, CA, USA) being used to generate figures. Demographic and work-related characteristics of participants were summarized using mean and standard deviations for continuous variables, and numbers and percentages for categorical variables. The validity and reliability of the domains used to access training needs were checked using Cronbach’s alpha as follows; quality management systems (α = 0.808), technical competence (α = 0.824), laboratory management, leadership, and coaching (α = 0.894), pathophysiology, data interpretation and research (α = 0.914). The Kolmogorov–Smirnov and Shapiro–Wilk tests were used to verify the normality of scale variables such as the ages of participants and their years in service. Differences in preference for training needs among groups were assessed by the Chi-square or Fisher exact test for categorical variables. Topics to be included in CPD programs to be developed were prioritized as follows: topics are given the most importance rating by ≥ 80% of the respondents (priority 1); 70–79% (priority 2); 60–69% (priority 3); and ≤ 59% (priority 4). Associations were explored between a set of indicators relating to demographic and work-related characteristics and preference for training needs using logistic regression models. *P* values less than 0.05 was considered statistically significant in all analysis.

## Results

### Respondent characteristics

This study received 328 responses; however, 12 responses were excluded because of missing and incomplete data. The online survey was accessed by 89 people (27% of the total number) in the first 24 h, and 187 (57%) by the end of the third day. A total of 316 responses that met the inclusion criteria and had complete data were analyzed for this study. The mean age of the respondents was 33.05 ± 6.33 with 250 (79.1%) being males, 63% being medical laboratory scientists, and 45.6% working with Ghana Health Service. The youngest respondent was 22 years old, while the oldest was 56 years old. The respondents had varying years of work experience, ranging from one year to over 30 years of experience. One hundred and forty-six respondents reported being in supervisory roles; 67 (45.9%) supervised 1–5 laboratory staff; while 45 (30.8%), and 34 (23.3%) supervised 6–10, and more than 10 laboratory staff, respectively (Table 1).

**Table 1:** Demographic and work-related characteristics of medical laboratory professionals recruited for the study

Variable	Categories	Frequency	Percentage
Age group (years)	20–30	131	41.5
	31–40	143	45.3
	41–50	38	12
	More than 50	4	1.3
Gender	Female	66	20.9
	Male	250	79.1
Cadre type	Medical laboratory assistant	10	3.2
	Medical laboratory technician	73	23.1
	Medical laboratory scientist	199	63
	Laboratory manager	31	9.8
	Others^a^	3	0.9
Current place of work	Ghana Health Service	144	45.6
	Mission hospital	74	23.4
	Teaching hospital	42	13.3
	Private hospital/laboratory	38	12
	Teaching/research institution	18	5.7
Years in service	1–5	143	45.3
	6–10	92	29.1
	11–15	45	14.2
	More than 15	36	11.4
Supervisory role	No	170	53.8
	Yes	146	46.2
Staff supervised	Non	170	53.8
	1–5	67	21.2
	6–10	45	14.2
	More than 10	34	10.8

^a^Others refer to professionals working with non-governmental organizations, regulatory, research, and training institutions

### Attendance of continuous professional development activities

**Fig. 1:**
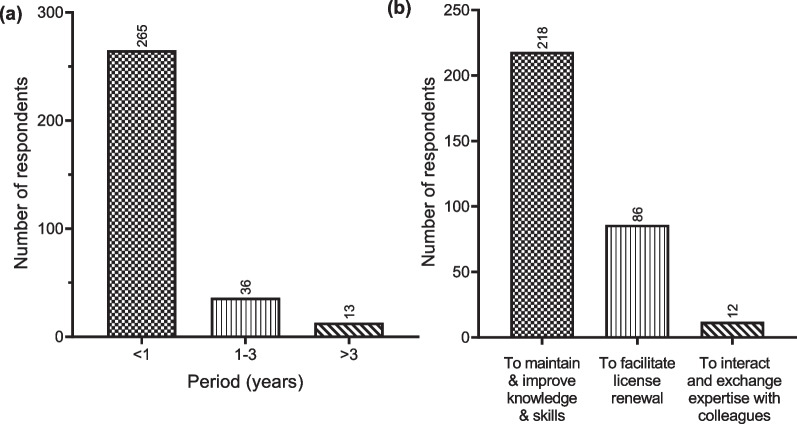
Responses provided by respondents on **a** the last time they attended a CPD program and **b** their major reason for attending CPD programs

**Fig. 2:**
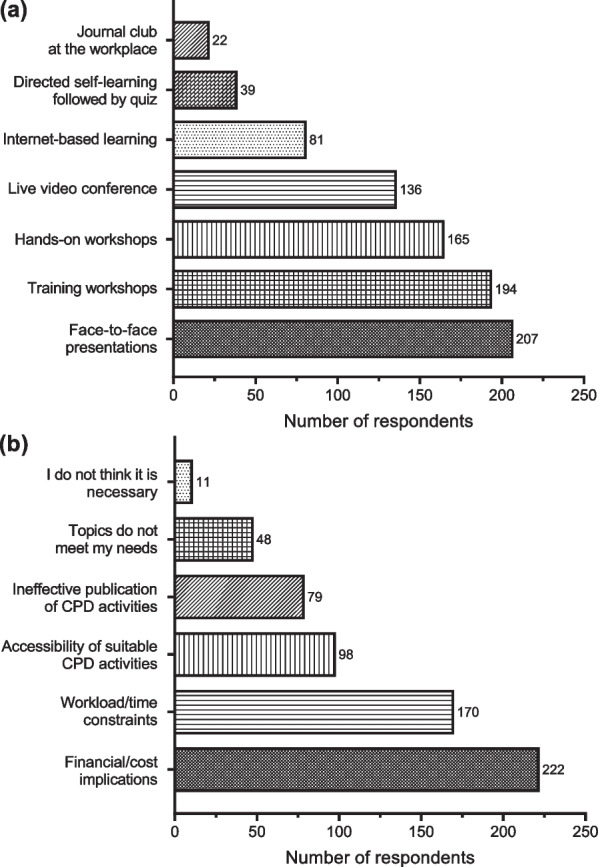
Responses provided by medical laboratory professionals on **a** preferred mode for CPD activities and **b** the major challenges impeding participation in CPD programs

When asked the last time they attended a CPD activity, the majority indicated that they had been part of a CPD activity within the last year, with a few indicating that the last time they participated in a CPD activity was between 1 and 3 years and more than 3 years (Fig. 1a). Most laboratory professionals attend CPD activities to maintain and improve their knowledge and skills. However, a few of them participate in CPD activities with the purpose of facilitating license renewal and exchanging expertise with colleagues in the field (Fig. 1b). The most preferred mode of CPD delivery by Ghanaian laboratory professionals is face-to-face presentations, training workshops, and hands-on workshops (2a). It is interesting to know that just a few laboratory professionals believe CPD is not necessary. However, most laboratory professionals cited financial implications and workload/time constraints as the main reasons impeding their participation in CPD activities (Fig. 2).

### CPD training preferences, ranking, and prioritization by medical laboratory professionals in Ghana

Table 2 details the ranking of selected knowledge and skills topics by medical laboratory professionals in Ghana. The results indicated that for the whole sample, all 47 items were rated most important by respondents with an average most important rating of 75.93 ± 9.21% (95% CI = 73.20–78.65) and a range of 70.3–82.6% suggesting that the respondents perceived themselves to have knowledge and skill deficits in all the topics and that these topics are essential for their practice. Overall, the most frequently selected topics for training based on domains for CPD training and ranking as most important were (i) quality management systems, (mean = 80.59 ± 9.024; 95% CI = 73.04–88.13); (ii) pathophysiology, data interpretation, and research (mean = 78.0 ± 6.973; 95% CI = 73.97–82.03); (iii) technical competence (mean = 73.97 ± 10.65; 95% CI = 66.35–81.59); and (iv) laboratory management, leadership, and coaching (mean = 72.82 ± 9.719; 95% CI = 67.44–78.2).

**Table 2:** Ranking of CPD training topics by medical laboratory professionals, *n* = 316

Training needs	Ranking			
	Most important *n* (%)	Moderate importance *n* (%)	Less important *n* (%)	No response *n* (%)
Quality management systems				
Quality system essentials for medical laboratory	274 (86.7)	31 (9.8)	5 (1.6)	6 (1.9)
Implementing a quality management system	277 (87.7)	31 (9.8)	4 (1.3)	4 (1.3)
Techniques to identify and control sources of errors in laboratory procedures	281 (88.9)	29 (9.2)	2 (0.6)	4 (1.3)
Management of non-conformances in laboratory services	196 (62.0)	104 (32.9)	11 (3.5)	5 (1.6)
Use of external quality assessment to improve testing procedures	247 (78.2)	53 (16.8)	8 (2.5)	8 (2.5)
Internal quality control and Westgard rule	253 (80.1)	51 (16.1)	4 (1.3)	8 (2.5)
Laboratory accreditation: principles and processes	237 (75.0)	68 (21.5)	8 (2.5)	3 (0.9)
Clinical laboratory safety	272 (86.1)	37 (11.7)	4 (1.3)	3 (0.9)
Technical competence				
Evidence-based laboratory medicine	273 (86.4)	34 (10.8)	2 (0.6)	7 (2.2)
Evaluation and selection of analytical methods and equipment	259 (82.0)	48 (15.2)	1 (0.3)	8 (2.5)
Definition, establishment, and use of reference ranges	245 (77.5)	61 (19.3)	5 (1.6)	5 (1.6)
Health informatics	193 (61.1)	103 (32.6)	11 (3.5)	9 (2.8)
Point-of-care testing	163 (51.6)	118 (37.3)	28 (8.9)	7 (2.2)
Statistics in laboratory medicine	221 (69.9)	81 (25.6)	5 (1.6)	9 (2.8)
Specimen management	254 (80.4)	49 (15.5)	3 (0.9)	10 (3.2)
Molecular diagnostic methods and genetics	235 (74.4)	66 (20.9)	6 (1.9)	9 (2.8)
Laboratory and disease surveillance	235 (74.4)	64 (20.3)	6 (1.9)	11 (3.5)
Equipment maintenance	259 (82.0)	42 (13.3)	6 (1.9)	9 (2.8)
Laboratory management, leadership, and coaching				
Customer care	259 (82.0)	44 (13.9)	4 (1.3)	9 (2.8)
Competence assessment	264 (83.5)	41 (13.0)	3 (0.9)	8 (2.5)
Ethics and professionalism	279 (88.3)	32 (10.1)	1 (0.3)	4 (1.3)
Supervision and delegation	239 (75.6)	65 (20.6)	3 (0.9)	9 (2.8)
Preceptorship and mentorship	206 (65.2)	92 (29.1)	9 (2.8)	9 (2.8)
Data management, report writing, and presentation skills	255 (80.7)	49 (15.5)	6 (1.9)	6 (1.9)
Basic cost accounting for clinical laboratory services	179 (56.6)	120 (38)	8 (2.5)	9 (2.8)
Management of resources and supplies	227 (71.8)	75 (23.7)	6 (1.9)	8 (2.5)
Monitoring and evaluation	230 (72.8)	72 (22.8)	3 (0.9)	11 (3.5)
Team building	248 (78.5)	48 (15.2)	8 (2.5)	12 (3.8)
Strategic planning	229 (72.5)	67 (21.2)	5 (1.6)	15 (4.7)
Rational selection of tests	215 (68.0)	86 (27.2)	4 (1.3)	11 (3.5)
Medical tariffs (billing and coding)	173 (54.7)	119 (37.7)	17 (5.4)	7 (2.2)
Cost of laboratory tests and procedures	202 (63.9)	92 (29.1)	13 (4.1)	9 (2.8)
Career prospects	247 (78.2)	54 (17.1)	6 (1.9	9 (2.8)
Pathophysiology, data interpretation, and research				
Case studies in clinical microbiology	265 (83.3)	38 (12)	4 (1.3)	9 (2.8)
Case studies in clinical chemistry	276 (87.3)	31 (9.8)	1 (0.3)	8 (2.5)
Case studies in hematology	275 (87.0)	30 (9.5)	1 (0.3)	10 (3.2)
Case studies in medical parasitology	260 (82.3)	45 (14.2)	1 (0.3)	10 (3.2)
Case studies in blood transfusion science	266 (84.2)	39 (12.3)	0 (0.0)	11 (3.5)
Case studies in cytology and histology	228 (72.2)	67 (21.2)	12 (3.8)	9 (2.8)
Case studies in immunology	261 (82.6)	39 (12.3)	6 (1.9)	10 (3.2)
Molecular and immunodiagnostic techniques	245 (77.5)	52 (16.5)	7 (2.2)	12 (3.8)
Semen analysis	250 (79.1)	49 (15.5)	7 (2.2)	10 (3.2)
Research proposal development and operational research	247 (78.2)	53 (16.8)	6 (1.9)	10 (3.2)
Grant proposal writing	222 (70.3)	73 (23.1)	10 (3.2)	11 (3.5)
Manuscript preparation	212 (67.1)	83 (26.3)	9 (2.8)	12 (3.8)
Basic computer skills	210 (66.5)	83 (26.3)	12 (3.8)	11 (3.5)
Use of statistical and programming tools	235 (74.4)	64 (20.3)	8 (2.5)	9 (2.8)

### Prioritization of training needs of medical laboratory professionals

We have used ratings based on the most important to prioritize training needs into four domains as detailed in Table 3. A total of 19 out of 47 training items have been prioritized as the number one need of laboratory professionals based on ranking as most important by more than 80% of respondents. Based on priorities, the three most important ranked individual topics were: techniques to identify and control sources of errors in laboratory procedures (88.9%), ethics and professionalism (88.3%), and implementing a quality management system (87.7%). The least ranked topics were: point-of-care testing (51.6%), medical tariffs (billing and coding) (54.7%), and basic cost accounting for clinical laboratory services (56.6%) (Table 3).

**Table 3:** Prioritization of CPD training topics by medical laboratory professionals in Ghana

Priority 1 ≥ 80%	Priority 2 70–79%	Priority 3 60–69%	Priority 4 ≤ 59%
1. Quality system essentials for medical laboratory 2. Implementing a quality management system 3. Techniques to identify and control sources of errors in laboratory procedures 4. Internal quality control and Westgard rule 5. Clinical laboratory safety 6. Evidence-based laboratory medicine 7. Evaluation and selection of analytical methods and equipment 8. Specimen management 9. Equipment maintenance 10. Customer care 11. Competence assessment 12. Ethics and professionalism 13. Data management, report writing, and presentation skills 14. Case studies in clinical microbiology 15. Case studies in clinical chemistry 16. Case studies in hematology 17. Case studies in medical parasitology 18. Case studies in blood transfusion science 19. Case studies in immunology	1. Use of external quality assessment to improve testing procedures 2. Laboratory accreditation: principles and processes 3. Definition, establishment, and use of reference ranges 4. Molecular diagnostic methods and genetics 5. Laboratory and disease surveillance 6. Supervision and delegation 7. Management of resources and supplies 8. Monitoring and evaluation 9. Team building 10. Strategic planning 11. Career prospects 12. Case studies in cytology and histology 13. Molecular and immunodiagnostic techniques 14. Semen analysis 15. Research proposal development and operational research 16. Grant proposal writing 17. Use of statistical and programming tools	1. Management of non-conformances in laboratory services 2. Health informatics 3. Statistics in laboratory medicine 4. Preceptorship and mentorship 5. Rational selection of tests 6. Cost of laboratory tests and procedures 7. Manuscript preparation 8. Basic computer skills	1. Point-of-care testing 2. Basic cost accounting for clinical laboratory services 3. Medical tariffs (billing and coding)

### Factors associated with CPD training need prioritization by medical laboratory professionals

The factors associated with the prioritization of training needs among the four domains of CPD included laboratory professionals’ current place of work, years in service, the reason for attending CPD activities, the period for attending the last CPD, supervisory role and number of staff being supervised (Table 4). Laboratory professionals who work in private hospitals and laboratories (OR = 4.38, 95% CI = 1.41–13.58) are more likely to prefer topics in quality management systems. Laboratory professionals who are working with Ghana Health Service (OR = 2.82, 95% CI = 1.12–7.09), teaching hospitals (OR = 4.64, 95% CI = 1.63–13.25), and private hospitals/laboratories (OR = 4.47, 95% CI = 1.50–13.32) would likely prefer topics on technical competence than colleagues working with teaching and research institutions. Laboratory professionals with 1–5 years of experience (OR = 3.42, 95% CI = 1.01–11.60) were more likely to prefer topics on laboratory management, leadership, and coaching (LMLC) than those with 15 years of experience. Laboratory professionals who attend CPDs for license renewal (OR = 0.54, 95% CI = 0.34–0.87) are less likely to prioritize topics in LMLC. Laboratory professionals who had not attended CPDs for more than 3 years (OR = 5.67, 95% CI = 1.39–23.14) and at Teaching Hospitals (OR = 3.12, 95% CI = 1.05–9.34) are more likely to prioritize topics in pathophysiology, data interpretation, and research (PDR). People in supervisory role (OR = 0.27, 95% CI = 0.11–0.65), and supervising 1–5 (OR = 0.39, 95% CI = 0.16–0.95) or 6–10 (OR = 0.39, 95% CI = 0.15–0.97) staff are less likely to prioritize topics in PDR.

**Table 4:** Factors that influence the choice of training needs of medical laboratory professionals in Ghana based on logistic regression analysis

Independent variable	Outcome variable							
	Quality management systems		Technical competence		Laboratory management, leadership, and coaching		Pathophysiology, data interpretation, and research	
	OR (95% CI)	*P*-value	OR (95% CI)	*P*-value	OR (95% CI)	*P*-value	OR (95% CI)	*P*-value
Age group								
20–30	0.52 (0.06–4.43)	0.547	0.28 (0.04–2.01)	0.207	0.22 (0.03–1.55)	0.129	0.16 (0.01–2.14)	0.167
31–40	0.42 (0.06–3.25)	0.407	0.26 (0.04–1.62)	0.148	0.29 (0.05–1.84)	0.191	0.16 (0.01–1.94)	0.150
41–50	0.53 (0.08–3.72)	0.522	0.69 (0.12–3.97)	0.680	0.67 (0.12–3.83)	0.653	0.13 (0.01–1.46)	0.099
> 50	1		1		1		1	
Gender								
Female	0.91 (0.53–1.57)	0.729	0.89 (0.54–1.49)	0.669	1.08 (0.64–1.83)	0.781	1.26 (0.73–2.15)	0.406
Male	1		1		1		1	
Cadre type								
Others	1.35 (0.13–14.43)	0.802	0.55 (0.05–5.99)	0.622	0.22 (0.02–2.01)	0.180	0.79 (0.07–8.44)	0.846
Lab Manager	1.82 (0.42–7.87)	0.423	0.69 (0.15–3.19)	0.634	0.58 (0.13–2.55)	0.470	0.54 (0.11–2.73)	0.457
Medical Lab Scientist	2.72 (0.76–9.71)	0.124	1.25 (0.32–4.930	0.750	0.95 (0.25–3.63)	0.936	1.26 (0.30–5.26)	0.755
Medical Lab Technician	2.18 (0.60–7.96)	0.237	0.70 (0.17–2.79)	0.609	0.78 (0.20–3.07)	0.724	0.92 (0.21–3.94)	0.905
Medical Lab Assistant	1		1		1		1	
Years in service								
1–5	0.50 (0.13–1.90)	0.307	2.15 (0.62–7.46)	0.228	3.42 (1.01–11.60)	**0.049**	1.56 (0.44–5.52)	0.492
6–10	1.04 (0.29–3.76)	0.958	2.24 (0.70–7.20)	0.176	2.72 (0.88–8.44)	0.084	1.72 (0.55–5.41)	0.353
11–15	1.11 (0.34–3.66)	0.866	1.89 (0.64–5.61)	0.250	2.25 (0.76–6.62)	0.142	1.02 (0.36–2.94)	0.965
> 15	1		1		1		1	
Current place of work								
Ghana Health Service	2.36 (0.90–6.14)	0.080	2.82 (1.12–7.09)	**0.028**	1.21 (0.48–3.06)	0.687	0.76 (0.31–1.85)	0.541
Mission Hospital	1.39 (0.50–3.80)	0.528	2.08 (0.78–5.59)	0.145	0.71 (0.27–1.91)	0.500	0.71 (0.27–1.83)	0.474
Teaching Hospital	2.82 (0.95–8.39)	0.062	4.64 (1.63–13.25)	**0.004**	2.48 (0.87–7.08)	0.089	3.12 (1.05–9.34)	**0.042**
Private Hospital/Laboratory	4.38 (1.41–13.58)	**0.011**	4.47 (1.50–13.32)	**0.007**	1.83 (0.63–5.32)	0.269	1.60 (0.55–4.68)	0.390
Teaching/Research Institution	1.00		1		1		1	
Being in a supervisory role								
No	0.52 (0.22–1.22)	0.133	0.74 (0.32–1.71)	0.481	0.56 (0.26–1.20)	0.133	0.27 (0.11–0.65)	**0.003**
Yes	1		1		1		1	
No. of staff supervised								
0	1		1		1		1	
1–5	0.60 (0.25–1.43)	0.252	0.96 (0.41–2.23)	0.923	1.02 (0.47–2.22)	0.960	0.39 (0.16–0.95)	**0.039**
6–10	0.42 (0.17–1.05)	0.064	0.49 (0.21–1.19)	0.115	0.65 (0.29–1.49)	0.311	0.39 (0.15–0.97)	**0.044**
> 10	1		1		1		1	
Period for last CPD								
Not specified	1.97 (0.51–6.22)	0.999	3.34 (1.12–9.87)	0.999	4.81 (0.36–63.90)	0.234	0.20 (0.01–3.82)	0.281
> 3	1.95 (0.60–6.32)	0.266	2.80 (0.83–9.47)	0.097	3.62 (1.19–11.00)	**0.023**	5.67 (1.39–23.14)	**0.016**
1–3	1.05 (0.53–2.09)	0.890	0.87 (0.47–1.63)	0.668	0.98 (0.51–1.86)	0.944	1.17 (0.61–2.26)	0.639
< 1	1		1		1		1	
Reason for attending CPD								
Facilitation of license renewal	0.69 (0.42–1.12)	0.133	0.67 (0.42–1.07)	0.096	0.54 (0.34–0.87)	**0.010**	1.10 (0.67–1.82)	0.696
Exchange of expertise with colleagues	0.55 (0.18–1.68)	0.297	0.77 (0.26–2.33)	0.647	0.72 (0.25–2.08)	0.539	0.38 (0.12–1.18)	0.095
Improvement in knowledge and skills	1		1		1		1	

*OR* odds ratio; *95% CI* 95 percent confidence interval

## Discussion

This study sought to identify and prioritize the training needs of Ghanaian medical laboratory professionals and highlight the challenges faced in accessing CPD programs. It is hoped that the findings of this study would help provide focused CPD that meets the needs of practitioners as well as the changing trends in medical laboratory practice. Laboratory professionals reported financial cost implications (67.7%) and workload/time constraints (51.8%) as the main reasons impeding their participation in CPD activities. Financial and workload constraints have been cited as major issues impeding CPD attendance by laboratory staff in Nigeria [19]. CPD attendance is hampered by a lack of relief coverage, inability to take paid or unpaid study leaves, the use of personal time for required training, inadequate staffing, and issues with leadership [20]. Given the changing financial situation in the country and around the world, CPD planners should target developing cost-effective events which could easily be assessed. Again, employers and supervisors should support the training of staff by providing clear policies to support staff CPD. CPD should be seen as a shared professional responsibility where organizations gain from better care for patients as staff develops themselves professionally [20].

The study identified (i) quality management systems; (ii) pathophysiology, data interpretation, and research; (iii) technical competence; and (iv) laboratory management, leadership, and coaching as the priority areas for training by laboratory professionals in that order. Similar domains have earlier been identified as priority areas for personnel in Botswana [3]. The study also reported quality management systems as the highest prioritized among their medical laboratory professionals. This similarity reflects the increased demand for total quality management within medical laboratories across Africa [21–23]. Contrary to the findings of the present study, healthcare professionals in Uganda cited research and audit as the domain with the largest training need [24] while health workers in the United Kingdom preferred topics that addressed anxiety and lack of confidence [25]. The training needs of health workers may vary by location. There has been an increased demand for quality management systems in the Ghanaian health sector as many medical laboratories strive for international accreditation. It was therefore not surprising that techniques to identify and control sources of errors in laboratory procedures (88.9%), ethics and professionalism (88.3%), and implementing a quality management system (87.7%) emerged as the topmost ranked topics. Knowledge in these areas is necessary for meeting international laboratory standards such as the International Organization for Standardization (ISO 15189), Clinical and Laboratory Standards Institute (CLSI), and Good Clinical Laboratory Practices (GCLP). Human resource is the organization’s most crucial resource whose behaviors, talents, and aspirations affect: the other resources that the organization uses, the organizational efficiency, and its effectiveness [26]; hence prompt policies and intervention in CPD programs should target addressing their training needs to help in promoting quality health care delivery in the country.

The most preferred modes of CPD delivery reported in the present study are face-to-face presentations, training workshops, and hands-on workshops. Our findings are quite similar to those earlier reported by medical laboratory professionals in Botswana [3] and among other health workers [27, 28]. These studies reported that training workshops, hands-on workshops, and internet-based learning were the most preferred mode of CPD training among medical laboratory professionals in Botswana. In other studies, the online mode was preferred by health workers and managers for CPD delivery [18, 29]. Contrarily, just a few medical laboratory professionals (24%) in our study population preferred internet-based learning. The possible reason for these modes of preferences may be due to familiarity with these methods ─ the traditional way of organizing training workshops has always been the case where medical laboratory professionals are required to travel and converge at designated centers. Aside from the training, it has always been an opportunity to reunite with colleagues, make new relations and explore other opportunities; this in our view, might be a key factor influencing the choices selected by the respondents. Due to advancements in wireless and smartphone technology, eLearning has switched to mobile learning, which is seeing a significant increase in use due to the COVID-19 pandemic [30]. However, the lack of stable internet services and insufficient skills in the use of information technologies could be reasons why most participants would rather not opt for internet-based platforms for CPD delivery.

### Strengths and limitations of the study

This study is the first survey of Ghanaian health professionals, as far as we know, that aimed to identify and prioritize what professionals report as their training and development needs. Responses were received from all representative regions of the country, and medical laboratory scientists in all cadres with varying years of experience. The findings of this study should be considered in light of some limitations; first, the lack of an accessible and comprehensive national registry of medical laboratory professionals in the country may have hampered the recruitment of participants. However, respondents’ different ages, varying years of experience, cadre, and places of work justify the representativeness and generalizability of the study. Although the online survey method of data collection employed limits potential interviewer bias and social desirability bias, participants may have misread or misunderstood certain items in the survey instrument. Lastly, as this study was conducted using a cross-sectional design, the results could not imply any cause–effect relationship as reverse causality remains a possibility.

## Conclusion

This study has identified what laboratory professionals in Ghana perceive as important training needs, the preferred mode of delivery, and challenges impeding their participation in CPD programs. Topics addressing quality management systems were identified as the most preferred by participants. The identified needs shall help in developing CPD programs that address what medical laboratory professionals perceive as educational and professional training needs and meets the changing medical trends. Ghanaian medical laboratory professionals cited face-to-face presentations, training workshops, and hands-on workshops as their preferred mode of CPD delivery, and financial implications and workload/time constraints as the main reasons impeding their participation in CPD activities. We recommend that stakeholders including the Allied Health Professions Council, Ghana Association of Medical Laboratory Scientists, accredited academic institutions, and organizers of CPD activities should incorporate these training needs into future programs and address challenges highlighted in this study. This would help in having a more focused and targeted continuing professional development that meets the needs of practitioners and clients as a whole.

## Abbreviations

CPD: Continuing professional development
OR: Odds ratio
CI: Confidence interval

## Declarations

### Availability of data and materials

All data generated and analyzed during this study are included in this published article and its supplementary information files (Additional file 1).
